# Endosymbiotic Evolution of Algae, Secondary Heterotrophy and Parasitism

**DOI:** 10.3390/biom9070266

**Published:** 2019-07-08

**Authors:** Miroslav Oborník

**Affiliations:** 1 Institute of Parasitology, Biology Centre CAS, 37005 České Budějovice, Czech Republic; obornik@paru.cas.cz; Tel.: +420-387-775-464; 2 Faculty of Science, University of South Bohemia, 37005 České Budějovice, Czech Republic

**Keywords:** endosymbiosis, evolution, plastid, photosynthesis, secondary heterotrophy, phagotrophy, parasitism

## Abstract

Photosynthesis is a biochemical process essential for life, serving as the ultimate source of chemical energy for phototrophic and heterotrophic life forms. Since the machinery of the photosynthetic electron transport chain is quite complex and is unlikely to have evolved multiple independent times, it is believed that this machinery has been transferred to diverse eukaryotic organisms by endosymbiotic events involving a eukaryotic host and a phototrophic endosymbiont. Thus, photoautotrophy, as a benefit, is transmitted through the evolution of plastids. However, many eukaryotes became secondarily heterotrophic, reverting to hetero-osmotrophy, phagotrophy, or parasitism. Here, I briefly review the constructive evolution of plastid endosymbioses and the consequential switch to reductive evolution involving losses of photosynthesis and plastids and the evolution of parasitism from a photosynthetic ancestor.

## 1. Introduction

Phototrophic organisms are the foundation of every food chain on Earth. We all depend on the ability of tiny bacteria to photosynthesize, using the energy from sunlight for the conversion of CO_2_ and water into organic compounds. Photosynthesis first appeared in cyanobacteria dated by fossil records to about 3.5 billion years ago [1]. Since there was no free oxygen in the atmosphere at that time [1], and because oxygen was toxic to all organisms living in pre-oxygenic times, cyanobacteria, as producers of free oxygen, caused one of the most devastating environmental disasters in the history of this planet. In various forms, either as free-living bacteria or in endosymbiotic associations with eukaryotes, phototrophic bacteria colonized the oceans and dry land. Since cyanobacteria were more or less limited to the aquatic environment, they entered into a mutualistic relationship with a heterotrophic eukaryotic cell in an endosymbiotic event dated about one billion years ago [2]. This led to the evolution of symbiotic entities in primarily phototrophic eukaryotes deeply integrated into the host cell in the form of eukaryotic organelles called plastids. Plastids are interpreted as symbiotic bacteria [3,4,5,6] or eukaryotic organelles [7,8,9,10,11] captured by eukaryotes directly in the prokaryote-to-eukaryote endosymbiotic event or as higher order eukaryote-to-eukaryote endosymbioses. The evolution of photosynthesis together with endosymbioses allowed for the expansion of phototrophic organisms across the planet. Photosynthesis transforms the energy of sunlight into fuel for heterotrophic life forms through the production of energetically rich organic carbon-containing molecules (sugars), with their subsequent decomposition by glycolysis and oxidative phosphorylation. This biotrophic energetic cycle enabled by the unique molecular machinery of electron transport chains in photosystems and respiratory chains facilitated the striking success of life on Earth. Both peerless types of molecular machinery evolved just once in evolutionary history and have been transferred to various eukaryotic life forms only through plastid and mitochondrial endosymbioses.

## 2. Primary Endosymbioses

**Figure 1:**
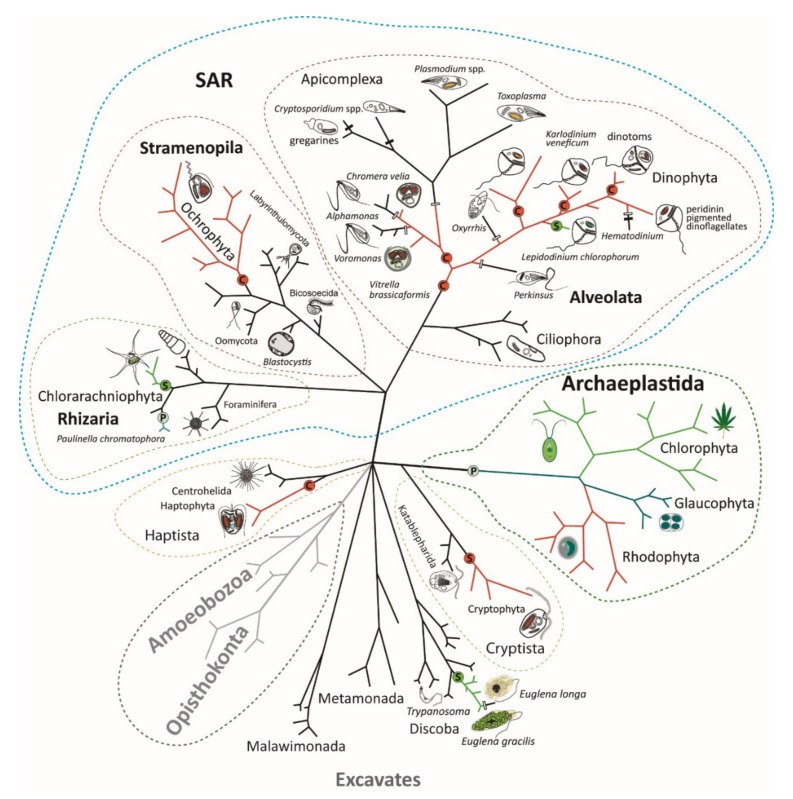
Diversity of eukaryotic phototrophs. Endosymbiotic events are shown in the hypothetical tree: P—primary endosymbioses; C—complex endosymbioses; S—secondary endosymbioses. Losses of photosynthesis (white rectangles) or losses of the entire plastid (black rectangles) are indicated. SAR: Stramenopila + Alevolata + Rhizaria.

Plastid primary endosymbiosis is an endosymbiotic relationship between phototrophic bacteria (cyanobacteria) and a eukaryotic host. It is believed that the ancient primary endosymbiosis leading to the evolution of primary plastids in Archaeplastida was a single event [12] (Figure 1). Such plastids possess a two-membrane envelope, which corresponds to the two membranes surrounding the ancestral cyanobacterium. It is believed that the ancestor of Archaeplastida then diverged into three lineages: Glaucophyta, Rhodophyta, and Chlorophyta. Glaucophytes are a small group of obscure algae possessing photosynthetic symbionts (organelles called cyanelles), which still have the remnant of the cyanobacterial cell wall [13]. Glaucophytes as well as rhodophytes (red algae) harvest light like cyanobacteria by phycobilisomes composed of phycobiliproteins. This type of light harvesting protein has been lost in green algae and plants (Chlorophyta). Primary eukaryotic phototrophs differ in their tetrapyrrole pigmentation. While glaucophytes are pigmented by chlorophyll *a*, rhodophytes use chlorophylls *a* and *d*, and chlorophytes use chlorophylls *a* and *b* [14,15]. 

Eukaryotic endosymbiosis is not just a simple engulfment of an endosymbiont. It is a complex process that also involves metabolic and genetic integration of the endosymbiont and host cell. Many genes have been lost from the symbiont during evolution, and about 1500 of them have been transferred to the host nucleus by endosymbiotic horizontal gene transfer (EGT) [8,16]. These genes are translated into the cytosol, but their products (proteins) are posttranslationally targeted to the plastid where they function [8,16]. Additionally, it has been shown that enzymes that were not originally cyanobacterial (plastid) can also contribute to plastid metabolism. For example, some enzymes of the plastid-localized heme biosynthesis pathway originate from the mitochondrion (porphobilinogen deaminase in chlorophytes and rhodophytes) [17,18], the eukaryotic nucleus (porphobilinogen deaminase in glaucophytes; uroporphyrinogen decarboxylase and coproporphyrinogen in Archaeplastida) [18,19], or even from bacteria, being obtained through non-endosymbiotic horizontal gene transfer (ferrochelatase in rhodophytes) [18]. Nuclear-encoded plastid-targeted enzymes are imported into the plastid by well-characterized translocon (TIC and TOC) machinery [8].

Although it is believed that the primary endosymbiosis leading to the evolution of glaucophytes, rhodophytes, and chlorophytes was a single event, this idea has been questioned by John W. Stiller and colleagues [20]. Functional and physiological constraints could have driven the loss of genes from plastid genomes in a similar direction because they faced roughly the same selection pressure from the host and analogous physiological requirements. Likewise, the same set of genes could have been retained to keep the plastids functional, such as housekeeping genes and genes for the proteins involved in photosynthesis. An analyses performed by Stiller and colleagues demonstrated that the similarity in the gene content among the plastid lineages is better explained by convergence than common ancestry. In spite of the attractiveness of this interesting consideration, at least when the gene content of plastid genomes is taken into account, the hypothesis claiming that a single endosymbiotic event created the ancestor of all three primary plastid lineages has much greater support [8,9,10]. The main reason for this is that the endosymbiotic process is so complex that it makes multiple primary endosymbiotic scenarios much less parsimonious [21]. In contrast with this assumption, a second case of an independent primary endosymbiosis between a heterotrophic eukaryotic host (the cercozoan *Paulinella chromatophora*) and a cyanobacterium was confirmed in 2005 [22]. This rhizarian hosts a phototrophic cyanobacterial symbiont with a genome reduced to approximately half that of its free-living ancestor. This endosymbiont keeps characteristics of the cyanobacterial genome, such as gene synteny. In the case of the *Paulinella* phototrophic symbiont (also called “cyanelle” or “chromatophore”), it is even possible, contrary to the plastids of plants and algae, to phylogenetically specify the particular taxonomic affiliation of the cyanobacterial ancestor [23]. The timing of this unexpected second primary endosymbiotic event was dated to between 60 and 140 MYA [24].

## 3. Complex Endosymbioses

When looking at the eukaryotic tree of life (Figure 1), it is clear that the diversity of eukaryotic phototrophs is much greater than the Archaeplastida and *Paulinella*, with their cyanobacterial phototrophic symbionts. Other eukaryotic supergroups also contain phototrophs: namely, the SAR group (Stramenopila + Alevolata + Rhizaria) and the paraphyletic assembly, referred to as Excavates, particularly euglenophytes. Within the SAR clade, all three subgroups—stramenopiles, alveolates, and rhizarians—contain phototrophic algae. Photosynthetic stramenopiles (also called ochrophytes) constitute a crown group with several early branching heterotrophs, such as Bicosoecida, Oomycota, Labyrinthulomycota, and *Blastocystis*. In the alveolates, there is a similar arrangement, with early branching heterotrophic ciliates. The vast majority of rhizarians are heterotrophic, but in the cercozoans, there are two photosynthetic algal groups: the aforementioned genus *Paulinella* and the chlorarachniophytes. Analogously, euglenophytes constitute the phototrophic crown group of euglenids. There are two additional phototrophic groups, haptophytes and cryptophytes, with unclear phylogenetic affiliations. Ultrastructural investigations have shown that the plastids of all the above-mentioned phototrophic organisms are, unlike the double-membraned primary plastids, equipped with a multi-membraned envelope, usually composed of three (euglenophytes and dinoflagellates) or four (ochrophytes, chromerids, apicomplexans, cryptophytes, haptophytes and chlorarachniophytes) membranes, with additional translocon machineries, such as the symbiont-specific ERAD (endoplasmic reticulum-associated degradation) -like machinery for plastid-targeting of nuclear-encoded proteins. It is believed that these plastids evolved through complex (secondary or higher order) endosymbiotic events involving a eukaryotic host and a eukaryotic phototrophic endosymbiont—an alga hosting a primary or complex plastid [7,8,9,10,12,25,26,27,28]. 

All secondary plastids identified to date originate from the endosymbionts of the two primary plastid lineages, rhodophytes and chlorophytes. There is a general consensus regarding the number of secondary endosymbiotic events that involved chlorophyte endosymbionts. Accumulating evidence shows that one such event happened in the ancestor of euglenophytes and the second in the ancestor of chlorarachniophytes (Figure 1) [10,12,25,27,28]. While the chlorarachniophyte plastid is surrounded by a four-membrane envelope and possesses a remnant of the endosymbiont’s nucleus called the nucleomorph [29], the plastid of euglenophytes is structurally reduced to a simple three membrane plastid, with no rudimental structures originating from the cytosol of the endosymbiont [30]. Remarkably, green secondary plastids can also be found in dinoflagellates. The genus *Lepidodinium* hosts a chlorophyte-derived plastid, which is believed to have originated in a serial secondary endosymbiosis [31,32]. This means that the dinoflagellate had a rhodophyte-derived plastid in its evolutionary history, which was replaced by the chlorophyte endosymbiont. In agreement with the “shopping-bag“ hypothesis [33], the green plastid uses some of the enzymatic equipment of the former red plastid inhabitant—for example, the enzymes used for tetrapyrrole biosynthesis [18,19]. 

Conversely, the number of eukaryote-to-eukaryote endosymbioses involving a rhodophyte or rhodophyte-derived endosymbiont is a subject of much discussion and is unlikely to ever be solved with certainty. At one extreme, Thomas Cavalier-Smith (TCS) proposed the placement of a single endosymbiotic event at the root of the hypothetical group called “Chromista”, involving all algae with rhodophyte-derived plastids, such as those in the SAR group (ochrophytes, dinoflagellates, chromerids and their relatives), as well as haptophytes and cryptophytes [34], because, due to its complex nature, the endosymbiotic establishment of an organelle is assumed to be an improbable occurrence. This concept enhances the Chromalveolate hypothesis published by TCS in 1999 [21] by including rhizarians, a group that has, however, never been shown to contain any rhodophyte-derived plastid. Since most of the groups of complex red algae contain early branching heterotrophs, such a scenario would require massive losses of plastids in at least five lineages: Haptista (including Haptophyta), with early branching centrohellids; Cryptista (including Cryptophyta), with early branching Katablepharida; Rhizaria (almost the entire group is heterotrophic); Stramenopila, with ancestral heterotrophic Oomycota, Labyrinthulomycota, Bicosoecida, and others; and Alveolata, with early branching Ciliophora (ciliates) (Figure 1). The proposal published by Paul Falkowski and coworkers represents the opposite extreme. It assumes that independent secondary endosymbioses were responsible for the appearance of all the groups of algae that possess a complex rhodophyte-derived plastid: peridinin pigmented dinoflagellates, cryptophytes, haptophytes, and ochrophytes (diatoms) [27]. Other published scenarios propose various combinations of secondary and higher order complex endosymbioses (tertiary, quaternary, etc.). Some of them [35,36,37,38] assume that only cryptophytes host a genuine secondary rhodophyte-derived plastid. This idea is also supported by the fact that the cryptophyte plastid contains a remnant of the nucleus of the engulfed rhodophyte [39]. However, the presence of the nucleomorph in the cryptophyte plastid can be explained in two ways, either by its slow evolution or by its recent acquisition. A scenario involving a recent acquisition, which cannot be rejected, would complicate the subsequent steps in the proposed scenarios, which is a series of endosymbiotic events (tertiary and higher order) involving the cryptophyte endosymbiont being engulfed by ancestors of various algal lineages—haptophytes and ochrophytes [35] or only ochrophytes [36,38]. A tertiary endosymbiosis involving the stramenopile endosymbiont has been suggested for chromerids and apicomplexans [35,40,41,42], for an entire group of alveolates [38], and for haptophytes [36]. Since the origins of particular plastids are not clear, I prefer to use the term “complex plastid” for the photosynthetic symbionts participating in eukaryote-to-eukaryote endosymbiotic events. It should be noted that all of these proposals at least agree on the higher order (probably tertiary) endosymbiotic origins of some dinoflagellate plastids, including those from dinotoms that host a diatom endosymbiont, *Karlodinium* species, with a haptophyte-derived plastid, and the genus *Dinophysis*, harboring cryptophyte kleptoplastids [12,32].

## 4. Secondary Heterotrophy and Parasitism in Algae

Frequent losses of photosynthesis have been inferred in many complex plastids [8,10,12,43]. Although the ability to photosynthesize was beneficial and most likely the primary reason for keeping the plastids in algae, it is not essential for the host cell and does have some disadvantages. Many organisms that have had photosynthesis in their evolutionary history have frequently lost it. Photosynthesis is a biochemical process of transferring the energy of light to that of a chemical bond. However, it causes the production of reactive oxygen species (ROS), which can heavily damage the cell [44]. Reversals to a heterotrophic lifestyle can be found throughout the diversity of phototrophs, from bacteria [45] and primary algae to complex algae [46]. Although non-photosynthetic algae with complex plastids can be found in most lineages of eukaryotic phototrophs, the myzozoans (dinoflagellates, chrompodellids, and apicomplexans) represent the most prominent examples. In particular, apicomplexan parasites, former algae with a non-photosynthetic plastid, which cause devastating diseases in animals and humans (malaria, toxoplasmosis), have been extensively studied [47]. Photosynthesis was also lost from some parasitic plants [48], free-living green algae such as *Polytomella* [49], parasitic green algae such as *Helicosporidum* [50,51], parasitic rhodophytes [52], and osmotrophic euglenophytes (e.g., *Euglena longa,* [53]). Furthermore, about half of the dinoflagellates are secondarily heterotrophic, many of them being phagotrophic (e.g., *Oxyrrhis marina*, [54]).

Although losses of photosynthesis are quite frequent among eukaryotic phototrophs [9,28,32,55,56], loss of the entire plastid is an extremely rare event. So far, only three such instances have been proven, all occurring exclusively in parasitic species. The plastid has been lost from apicomplexan parasites of the genus *Cryptosporidium* [57] and the closely related gregarines [58], as well as parasitic dinoflagellates of the genus *Hematodinum* [32,59,60]. Relic non-photosynthetic plastids are responsible for the synthesis of various compounds that are indispensable for the host cell and thus cannot be lost with impunity, except in parasites that scavenge these essential compounds from their host organism. Nonetheless, loss of the plastid is hypothetically possible for free-living organisms, particularly in the early stages of the endosymbiotic process, before any of the essential pathways are lost from the host cell (i.e., before any functions are delegated to the organelle). Admittedly, in such cases, we are unlikely to find any traces of the historical endosymbiosis or the plastid in the former host [43].

Many phototrophic eukaryotes are mixotrophic, combining phototrophy with a heterotrophic lifestyle [61]. Mixotrophy is frequently utilized in biotechnology for the production of lipids by algae, such as *Euglena gracilis* [62] and *Chlorella vulgaris* [63], and also by diatoms [64]. Most algae use osmotrophy as the heterotrophic method for acquiring organic carbon from their environment. However, other heterotrophic strategies are also used to support phototrophic organisms, particularly predation and parasitism. Predation is quite frequent among dinoflagellates, which can feed on diverse prey, including various bacteria, picoeukaryotes, nanoflagellates, diatoms, other dinoflagellates, heterotrophic protists (e.g., ciliates or free-living kinetoplastids), and even metazoans [61,65]. Mixotrophic predators usually use phagocytosis to capture their prey, an ability that can be found in many groups of algae with complex plastids, such as haptophytes (e.g., *Prymnesium parvum*), dinoflagellates (e.g., *Karlodinium armiger*, *Alexandrium pseudogonyaulax*, *Prorocentrum minimum*), and others. Surprisingly, in dinoflagellates, it is unlikely that predation is initiated by the need for organic carbon, as a substitute for the photosynthetic acquisition of this element, because prey intake is stimulated by higher photosynthetic activity. Predatory mixotrophic dinoflagellates hunt to obtain other essential nutrients, such as nitrogen and phosphorus.

In addition to the previously discussed trophic modes, many myzozoans live as parasites. Apicomplexa are the most prominent parasites in this group [47]. A majority of these protists contain the non-photosynthetic relic plastid (also termed the apicoplast) [66,67] of a complex rhodophyte-derived origin. Apicomplexans are thus highly modified algae, which have lost photosynthesis but retained the plastid. The relatively recent discovery of chromerids, photosynthetic algae closely related to apicomplexan parasites [40,68], provides a powerful tool for investigating the evolutionary transition from a photosynthetic alga to an obligate parasite. Sequencing of chromerid genomes has shown that there was massive gene loss in the ancestral apicomplexan parasite. The ancestral apicomplexan lost over 3800 genes during the transition from a phototrophic predecessor. On the other hand, only approximately 80 novel genes were gained. This may suggest that genes used for the phototrophic lifestyle have been adopted and modified for parasitism [69,70]. Chromerids possess some molecular features that are also found in colpodellids and apicomplexan parasites, such as the components of the heme pathway. In all these groups, the first common precursor for the heme pathway, δ-aminolevulinate (ALA), is synthesized by the C4 pathway from glycine, which is a pathway that is otherwise only found in eukaryotic heterotrophs and α-proteobacteria. This compound is then imported into the plastid, where either the next four steps (apicomplexan parasites) or the rest of the pathway (chromerids) takes place. This metabolic curiosity (all other phototrophs use the C5 pathway for ALA synthesis, with glutamate as the primary substrate) qualifies chromerids as the only known phototrophs that synthetize chlorophyll from glycine [18,71].

The scenario for apicomplexan evolution suggested by Geoffrey McFadden involves a transition from a symbiotic (mutualistic) phototroph in corals (such as *Chromera* velia) to a parasite [72]. This hypothesis was inspired by the assumption that chromerids are coral symbionts similar to dinoflagellates of the genus *Symbiodinium* [68]. However, all attempts to find the chromerids *C. velia* or *Vitrella brassicaformis* inside adult corals have so far failed [73,74]. This has led to the suggestion that both algae are more likely epiphytic, growing on the coral’s surface, rather than living as intracellular symbionts. However, Australian researchers have found *C. velia* inhabiting larvae of the stony coral, *Acropora digitifera* [75]. In addition, a recent investigation showed that the transcriptomic profile of coral larvae invaded by *C. velia* is similar to that of coral larvae infected by bacterial pathogens, suggesting that the chromerid is very likely a parasite, rather than a mutualist or commensal [76]. The mixotrophic combination of phototrophy and parasitism is quite rare in protists. As far as I know, apart from *C. velia*, it has only been described in the dinoflagellate, the *Blastodinium* sp, the photosynthetic parasite of copepods [77]. Photosynthesis is functional in this case, because the host is transparent to light. In contrast, ectoparasitic plants combine phototrophy and parasitism quite frequently [48]. The chromerid *C. velia* is probably a facultative or even an accidental parasite of coral larvae, and since no infected coral adults have been found so far, the infection is likely lethal for the larvae, or they cannot just continue to develop into a coral colony. In light of these findings, an evolutionary scenario from symbiosis (mutualism) to parasitism is unlikely. Mutualism requires a much more balanced metabolic relationship and deeper integration of the participants than parasitism. It is also believed that most ancestors of symbiotic bacteria were ancestrally pathogenic [78]. The recently discovered apicomplexans inhabiting corals named “corallicolids” still contain four genes of the chlorophyll synthesis in their plastid genome, although they do not synthesize chlorophyll. These enigmatic organisms may represent an intermediate stage in the transition from phototrophy to parasitism. However, their function in the coral and the type of interaction with the host is unknown [79]. The trophic mode switch from mixotrophy, combining phototrophy and parasitism in a light transparent host, to obligate parasitism seems to be more parsimonious. The loss of photosynthesis did not precede parasitism. Rather, it was a consequence of it, at least in the lineage of apicomplexan parasites.

## 5. Conclusions

The evolution of phototrophic eukaryotes was enabled by endosymbioses between a eukaryotic host and a prokaryotic or eukaryotic endosymbiont. While the prokaryote-to-eukaryote endosymbiotic events involving a cyanobacterial symbiont are believed to have happened twice during evolution, complex eukaryote-to-eukaryote endosymbioses are much more frequent. Since the main benefit of photoautotrophy is not essential for the survival of cells that have kept their ancestral mechanisms of heterotrophy (osmotrophy or phagotrophy), frequent losses of photosynthesis are found throughout the diversity of eukaryotic phototrophs. At the same time, a loss of the organelle is quite rare due to its acquired indispensability, as there were gradual losses and a redistribution of redundant pathways between the symbiont and the host. Many former phototrophs reverted to heterotrophy, obtaining organic carbon through osmotrophy, phagotrophy, and even parasitism. Apicomplexans are some of the most well-known parasites that have evolved from a photosynthetic alga. Contrary to the assumption that parasitism evolved from a mutual relationship, I suggest that a much more likely scenario involves a switch from mixotrophy (in the form of a combination of parasitic and phototrophic lifestyles) in the ancestral apicomplexan to obligatory parasitism. This scenario likely began in a transparent host (like it is now for *C. velia* and coral larvae), with the parasite losing its ability to photosynthesize when it invaded an opaque host, or as a consequence of scavenging all of its required organic carbon from the host.
